# Fevers

**Published:** 1846-10

**Authors:** 


### PART II.—REVIEW.

### ARTICLE IV.

*Fevers. Their Diagnosis, Pathology and Treatment.* Prepared and edited, with large additions, from the essay on Fever, in Tweedie's Library of Practical Medicine, by MEREDITH CLYMER, M. D., Prof. of the Principles and Practice of Medicine, in the Franklin Med. College, Philadelphia, etc. etc. Philadelphia: Lea & Blanchard. 1846. pp. 604. 8 vo.

Dr. Clymer has added to these essays an entire chapter on Typhoid Fever, and other valuable additions, amounting, as he informs us in the preface, to about one-half the volume; and which relate principally to the fevers of the country.

There are thirteen chapters, which comprise, The General Doctrines of Fever, Continued Fever, and Hectic Fever, by Dr. Christison. Typhoid Fever, by Dr. Clymer. Plague, Yellow, Intermittent and Remittent Fevers, by Dr. Shapter. Infantile Gastric Remittent and Puerperal Fevers, by Dr. Locock. Small Pox, by Dr. Gregory, and Measles and Scarlet Fever, by Dr. George Burrows.

The subject of Fevers being at this period one of much interest to every practitioner, we shall transfer to our pages some of Dr. Clymer's and the associated writers' views which are applicable to the fevers of the season.

The following extract, quoted by the editor from Professor Alison, points to the true cause and pathology of congestive fever, and through them, to the proper treatment.

"Even the peculiarities of that form of fever which has been described under the name of *Congestive*, are not to be explained by the mere circumstance of internal congestion, the existence of which, in the vessels, and especially in the veins of internal parts, in these circumstances, is admitted. For although congestion or stagnation of blood within the cranium may be held to be a sufficient cause of stupor, yet we are so far from regarding congestion in the great veins leading to the heart as a sufficient cause for deficient action there, and consequent feeble pulse and cold skin, that we have already stated the accumulation of blood in the great veins to be apparently the chief cause of the *increased* action of the heart, or the *reaction*, in the more usual form of fever. In the cases, therefore, where the congestion in the great veins fails to excite this reaction in the heart, some peculiar cause must have operated to prevent the heart from being usually excited, by the application of the unusual quantity of its natural stimulus; *i. e.*, the circumstance of unusually great and permanent congestion in the great veins, in the commencement of fever, is in all probability the effect, not the cause, of a peculiar sedative influence affecting the vascular system in these cases; such an influence naturally leading to accumulation of blood in the great veins, for the same reason that determines the accumulation there after death.

"That congestion of blood in the great veins is not *per se* adequate to account for the phenomena of any form of fever, appears distinctly from the fact, that no form of fever follows the great congestion there in cases of suspended animation in syncope, or from extreme cold, or submersion in water."

Dr. Clymer has chosen that classification, which every physician in his practice adopts despite all systems of Nosology.

"The simplest arrangement, and perhaps the best for practical purposes, is that founded on the peculiar phenomena which are constantly presented by the different forms of fever, constituting the types. In one variety, we have the febrile phenomena interrupted absolutely or incompletely at certain periods; whilst in another, the train of phenomena proceeds in an uninterrupted series; and a third is accompanied with a peculiar and characteristic eruption. The order that we shall observe, therefore, will be:

I. Continued Fevers.
II. Periodical Fevers.
III. Exanthematous or Eruptive Fevers."

He has given us a brief practical description of Ephemeral Fever, which may perhaps account to the minds of some for fevers "broken up" after the first exacerbation. And which until reminded of the existence of such a form, they have considered as Remittents.

"*Diagnosis.*—It is often extremely difficult to decide, at the outset of an attack, whether it is a case of ephemeral, periodic, or continued fever. The absence or presence of the causes just enumerated, may assist our inquiry. Whilst the non-occurrence or insignificance of the initial chill, and the continuation of the fever beyond six or eight hours will often enable one to distinguish it from intermittent fever; the amount of vascular excitement, the slight depression of the nervous powers, and the very transient duration of the premonitory symptoms will serve to distinguish it from the more serious varieties of fever."

In the chapter on Continued Fever, Synocha, Synochus and Typhus are treated of. The last will be read with interest in many of the more northern portions of our section of country, by those who think with Dr. Clymer that Typhus and Typhoid Fever are not essentially different.

He gives a table of the maximum frequency of the pulse in 181 cases of Eruptive Typhus, in which the average maximum of 90 cases in the male is 107,5, and in the greatest number of instances (20) is at 96. The average in 91 cases in the female is 114,1, and in the greatest number of instances (23) is at 108. The average of male and female is 110.8.

We insert Dr. Christison's account of the petechiæ in Typhus, not only because it is valuable and interesting in itself, but also that there have been seen in some portions of the country cases of a Remittent Type, in which petechiæ of the 2d variety made their appearance. It has not been observed that Typhoid symptoms were developed in these cases which received appropriate treatment.

"Three kinds of eruptions have often been comprised under the generic term petechiæ:—1. One, which is exceedingly rare, but which is occasionally remarked in the advanced stage of bad synochus or typhus for a short time before death, consists of small, pale brown, lenticular spots, without any elevation or roughness of the skin, and much resembling freckles. 2. Another, which is very common in some epidemics, and especially where the early stage of fever presents the inflammatory character, forms small, dark, reddish-black, roundish, accurately circumscribed, and often closely crowded spots, without elevation of the skin, and much resembling fleabites. Their resemblance to fleabites is such, that on the one hand, the latter are often mistaken for petechiæ; while on the other hand, some physicians will insist that real petechiæ are nothing else but fleabites. The two appearances, however, cannot be mistaken by a careful observer, because the petechial spot does not present the little dark point in the centre, which may be invariably seen in the fleabite, either with the naked eye, or with the help of a common magnifier. Sometimes the petechiæ are few in number, and readily escape notice; in other instances, on the contrary, they are excessively crowded. Their usual seat is upon the breast, shoulders, forearms, and legs; but they may be seen also on all other parts of the body except the face. They generally make their appearance towards the close of the first or beginning of the second week, and certainly not on a specific day, like the eruptions of the febrile exanthemata, as some have maintained. They are observed to occur chiefly in severe cases, but, from frequent observation in the epidemics of Edinburgh, they do not necessarily indicate danger; on the contrary, the cases in which they appear have proved rarely fatal. The appearance is owing to a thin stratum of extravasation on the surface of the true skin, and appears connected with increased force of the circulating system, being most characteristic where reaction is high. This form of petechial eruption has become rare (1838) for a few years past. 3. The third variety presents more or less numerous spots, of a paler, rather lake-red or rose red tint, irregular in shape, not distinctly circumscribed, but rather diffuse round the edge, with sufficient elevation of the skin to impart a sense of roughness to the finger, when drawn over a part where they are numerous. They present some resemblance to measles; and at times are so like that eruption, that the other symptoms must be looked to for the diagnosis. They present the same variety in number with the dark circumscribed petechiæ; they are usually most abundant over the chest, shoulders, forearms, legs, loins, flanks, and abdomen; and they are not unfrequently found loosely scattered round the loins, flanks, and upper part of the belly, although not visible anywhere else, so that, if not sought for, they may escape notice altogether."

Our limits do not permit us to make extracts from the treatment of Continued Fever, which with the matter added by the editor, will be found very full and practical; but pass to his chapter on Typhoid Fever, which has been written specially for the Middle and Northern States, but which will be equally applicable to some localities in the North West. The authorities referred to are principally American.

In regard to the unsettled question of the identity of Typhus and Typhoid Fevers, the author says:—

"The question of the identity of typhus and typhoid fever has been one of great interest, and respecting which much diversity of sentiment at one time prevailed. Whilst the British physicians held, with great unanimity, the opinion that, though different varieties, they were the same species of fever; the French and American authorities proclaimed their essentially dissimilar nature. For obvious reasons the present work is not suitable for the discussion of this point; and it is our intention merely to state the grounds on which those who contend for the non-identity of the two diseases base their opinions, and to ascertain how far they are in accordance with the observed facts. The leading features of difference insisted on to prove that the two varieties of fever—typhus and typhoid—are radically distinct, are, 1. The character of the eruption, which in typhoid fever consists of minute lenticular papulæ, disappearing on pressure, and thinly scattered over the abdomen and chest; whilst in typhus the spots 'are more irregular in their shape and size; not elevated above the adjacent skin; partially disappearing on pressure, or not at all; often abundant, or even confluent; in many cases occupying the skin of the extremities as well as that of the entire trunk, and usually of a duller and more dusky colour than in the former disease.' 2. The infrequency of the abdominal symptoms—diarrhœa, and pain and gurgling on pressure in the right iliac region—in typhus, which occurs so constantly in typhoid fever; and, 3. The absence in typhus of the characteristic intestinal lesions—specific alteration of the agminate and isolated follicles—of typhoid fever.

Let us briefly examine how far these asserted differences are sustained by observation, assuming, for the purpose of having some distinctive or pathognomonic trait assigned to typhoid fever, that a diseased condition of the glands of Peyer is necessary to constitute the disease."

To prove the opposite, he refers to Dr. Gaultier de Claubry, Dr. Landouzy, Dr. Felix Jacquot of France, Dr. John P. Mettauer of Virginia, Dr. Thomas Carroll of Cincinnati, his own experience in the Philadelphia Hospital, in Dublin and in London, from which he arrives at the subjoined conclusions.

"From these facts—few, it must be acknowledged, yet positive as far as they go—it is evident, 1. That there is no necessary or constant connection between the kind of eruption, and the abdominal symptoms or lesions;—that the lenticular eruption of the Paris fever, and the maculated, measly eruption of the Irish typhus, may exist conjointly with the diarrhœa, and pain and gurgling in the ileo-cœcal region, and with enlarged or ulcerated patches of Peyer. And 2. That there is no constant relation between the abdominal symptoms—pain, gurgling, and diarrhœa—and the intestinal lesions, since all these symptoms may be absent, not only in individual cases, but in a portion, or in the whole of those attacked in the course of a given epidemic.

"We would inquire whether the group of symptoms known as typhoid fever is perfectly identical at all times and at all places; aud further, if the essential characters of typhus are so constant or defined, that it can be taken as a fixed term of comparison? Do not epidemics of typhoid fever differ in their prominent phenomena, constantly, from sporadic cases, as well as from each other? Are there not a number of accessory circumstances, so subtile as to escape observation, perpetually modifying the influence of causes on the organism? General conclusions should not be drawn from limited observations, or from witnessing any disease so generally prevalent as the one under consideration, in a single district, or even country, and during a limited period of time. And we cannot avoid thinking that those who regard these two forms of fever as distinct and separate *species*, are premature in the expression of an opinion upon a subject in which there are, as yet, wanting many essential elements to warrant a positive judgment."

The chapters on intermittents and remittents contain much that is very interesting, particularly, those portions which give an account of the congestive or pernicious forms, which under various modifications have been so fatal in various parts of our Western country. Dr. Shapter's modification of intermittents generally are the inflammatory, congestive and malignant, we give his description of the latter two.

"The *congestive* form of ague is throughout of adynamic character. The cold stage, which is much protracted, is ushered in by vertigo, deep-seated pain of the head, followed by general trembling rather than rigor. The pulse is small and weak, and not unfrequently faintings and coma add to the alarm. The hot stage struggles on slowly, and, as it were, unwillingly, and then is but imperfectly developed; so that, instead of the usual characters of this stage, there is only a low oppressed condition. The sweating stage is scarcely perceptible. The period of intermission is marked by a pale, worn, contracted countenance, general oppression of the system, constricted and anxious breathing, and small, hard and frequent pulse. The surface of the body is colder than usual, with an incapacity of retaining the surface warmth at the same time that the internal parts feel heated and irritable. This modification of ague, however, seldom occurs, excepting in hot countries, where there is much prevailing marsh exhalation, and then only in those constitutionally nervous and irritable, or whose health has been impaired, and the powers of the system exhausted by previous disease. BOISSEAU states, that it occurs in quotidians, double tertians, tertians, and quartans; it sometimes takes on alternately these different types, whilst at other times they are irregular. (*Pyrétologie Physiologique.*) The duration of the congestive intermittent is but little known: it occasionally succeeds the adynamic continued fever; though, more frequently, it passes into the continued form. It is a peculiarly fatal variety of ague.

The *malignant* form of intermittent fever has been particularly described by ALIBERT. (*Traité des Fièvres Pernicieuses Intermittentes.*) After the second, third, or fourth accession of the febrile paroxysms, the cold stage becomes either shorter and more intense, or else very much prolonged; and in place of the phenomena usually attendant on the hot stage, urgent symptoms, hitherto not observed, show themselves; or those which had already characterized this stage are much exasperated. The sympathetic phenomena which specially characterize the febrile accession become less apparent, or cease almost entirely, while symptoms of local irritation, hitherto unperceived, become developed. Nevertheless, the paroxysm passes off without any very well-pronounced perspiration, but a fetid odour is often exhaled from the body. The patient in part recovers his powers and appetite, and sometimes even does not complain of any particular uneasiness. On the accession of the succeeding paroxysm, however, colliquative hemorrhages and petechiæ often make their appearance; and not unfrequently death ensues at this period, or the disease may be protracted to the third, fourth, or fifth paroxysm.

Such is the outline of what French writers have termed *Fièvres intermittentes pernicieuses:* they usually occur in warm climates in persons of broken-down constitution, as well as when the intermittent fever is complicated with organic."

Dr. Clymer has to this added the annexed description of a similar form of fever which we give entire.

"The subject of the pernicious forms of intermittent fever is one of great interest to the practitioners of an extensive district of this country, where these varieties prevail to a greater or less extent. Those forms to which the term *pernicious* is applied, are, in reality, cases characterized by the greater violence of the accompanying congestions, and where, from the importance of the organs implicated, death is imminent at the third or fourth accession.

"A highly interesting account of an epidemic ague, of the *pernicious* variety, occurring in Persia in 1842, has been given by Dr. CHARLES W. BELL, Physician to the British Mission. It was essentially a quotidian ague, characterized by intense general congestion of the venous system. The disease had several modes of commencement. 'Sometimes it began at once, by the patient becoming suddenly insensible without previous symptoms; at other times it was preceded by formal ague. In many instances, again, the patient would suffer for some time previously from intermitting headache, daily increasing, and great want of sleep; he would then have one attack of ague, and, next day, at the same time, would sink down insensible. This was the form of disease from which the greatest number of deaths took place, and obtained for the malady its Persian name TAB-I-GHASH, or "fainting fever." During the insensibility the pulse was feeble and the extremities cold. From this state many were never roused; but if they were, the pulse gradually attained power, and the patient came slowly to his senses, complaining of intense headache and a feeling of oppression at the heart; a low kind of fever then came on which was succeeded by very imperfect perspiration, generally confined to the head and chest. Next day, about the same hour, insensibility returned, and each attack continuing longer than the preceding one, the period of death depended upon the strength of the patient or violence of the disease; most frequently, however, death took place on the third attack. As the end approached the secretion of urine ceased, the efforts of the heart at reaction became feebler, the skin felt like that of a corpse, cold and damp, the body became purple and mottled, and the pulse became less perceptible at the wrist: at length the patient was seized with tetanic conculsions and died. In these cases, as often observed in cholera, the feet began to get warm shortly before death, and just as the warmth had spread up the legs and reached the trunk the patient died. Indeed, were other symptoms wanting, I should consider warmth commencing in the feet while the rest of the body was cold, quite sufficient to mark the case as hopeless." In another form, resembling the *algid* of southern countries, there was 'no insensibility, no shivering, little or no perceptible fever, and no perspiration; the primary characteristic symptoms were a fixed pain in the pit of the stomach, extreme tenderness on pressure over the left lobe of the liver and region of the spleen, and extreme tension of the abdominal muscles. One or both of the recti abdominis became hard as a board, continuing for days in a state of constant tension, but without any painful cramps or spasms. Nearly at the same hour each day the patient was observed to become exceedingly anxious and restless, tossing from side to side, sighing and throwing the arms above the head, as in yawning, and the pulse became very small and frequent, and the body damp and cold. By and by, this oppression passed off, the body resumed its natural warmth, and the pulse nearly its natural volume; but this continued quicker than usual, and then to all appearance the patient had very little the matter with him. Each day, however, the oppression of the circulation became greater and the attack continued longer; the pulse now became weaker, and an ice-cold exudation ran off the brow and back of the hands. The struggling of the heart to overcome the load of blood which oppressed it was most painful to listen to,—now almost overcoming the obstruction, the pulse for an instant gaining power, and a partial warmth spreading over the surface; and, again, the force of the heart succumbing to the disease, and the icy coldness—much colder than death—returning. The craving for iced water was incessant so long as this state lasted. The evacuations meantime were bilious, and the quantity of urine daily diminished, and at length ceased altogether. At length the intermission between the attacks of oppression ceased to occur, the pulse was only perceptible at intervals, and the patient, who up to this time had been perfectly sensible and even able to walk to stool, fell into a state of stupor. The skin now became blue and mottled, and the patient gradually sunk or died in convulsions. Here, also, as I remarked above, some time before death took place, the lower limbs recovered almost their natural warmth. In all the cases I saw of this variety there was much feeling of distension of the stomach and inactivity of the bowels, and sometimes a little vomiting.' The great and rapid enlargement of the spleen is particularly mentioned. In many instances pain in the region of the spleen was felt before the occurrence of any other symptom. The blood was of a dark, dusky, reddish-brown colour, and in general the serum did not separate from the clot. In those affected with the severer forms of the disease, the blood drawn in the cold fit was always grumous, coming at first slowly or in drops, and coagulating as soon as drawn, even at the mouth of the wound; and no separation of the serum took place. During the epidemic the urine of the people in general was much darker colored than at other times, while in those who were seriously affected it was, if secured at all, like porter, and in very small quantity."

The treatment recommended for these forms of intermittents is the one that has been found to be the most uniformly successful, and of which accounts have from time to time been published in the American Journals.

"In the congestive form vigorous practice is urgent. Maillot gives an example in which 40 grs. of the sulphate of quinine and 2 drachms of ether were given in 4 oz. of water at two doses, in the course of an hour; a starch opiate injection, with 60 grs. of the sulphate of quinine and 2 drachms of ether, was ordered at the same time, with sinapisms to the legs and blisters to each thigh. Under this treatment the patient began to recover warmth in a few hours, and the heart to act more forcibly; but the next morning the amendment was so slight, that a sinapism was applied to the whole length of the spinal column, and a clyster with one drachm of sulphate of quinine, and three drachms of ether administered; reaction followed with recovery. Every effort must be made to produce speedy reaction. Stimulants should be freely given—

brandy, ammonia, and particularly capsicum—both by the mouth and rectum; bottles of hot water, and hot bricks are to be applied to the extremities; sinapisms to the trunk and extremities, with turpentine fomentations to the chest and abdomen. As the pulse becomes developed, this active and violent treatment must yield to milder stimulants and diaphoretics. Quinine should be administered freely in large doses, by the stomach and in enemata, and it may also be rapidly introduced into the system through blistered surfaces, produced by the application of ammonia, and by inunction."

Dr. Clymer has also added to remittent fever an account of two fatal varieties, which will complete the description of the pernicious forms.

"A variety of pernicious remittent, is occasionally met with in our southern states, which may be called the *comatose* form, and which resembles closely the same variety of pernicious intermittent already described. The force of the malarious poison seems in such cases to be expended on the great nervous centres. Commencing with slight shivering, the vascular reaction soon becomes intense; the face is full, and flushed; the pulse firm and full, there is strong pulsation in the larger arteries, especially the carotids; deep stupor soon comes on, with dilated pupils, and slow and often stertorous breathing. As the paroxysm abates the stupor subsides, and during the remission, which generally lasts but for a few hours, no alarming symptom is present. On the return of the paroxysm, which frequently anticipates itself, the same train of symptoms appear with increased severity. And this is repeated until recovery or death occurs. The remissions are sometimes very imperfect, and Dr. Boling relates a case of this kind where the patient lay eight days comatose. When called to him, he says, 'he presented all the symptoms of apoplexy, and nothing revealed the true nature of the case but a disposition to yawn and stretch every morning, continuing from 7 A. M. to 10 A. M., and a slight abatement in the force, and a diminution of a few beats in the frequency of the pulse, with a temporary disappearance of the stertor. During the remissions, while yawning and stretching, his appearance was exactly that of a person just on the point of awaking from a sound and refreshing sleep, and the bystanders, even those who had seen him several times, could scarcely divest themselves of the impression that this was the case, and were in momentary expectation of seeing him open his eyes and address them. The case terminated favourably, the patient waking up during the hour of remission on the 9th morning, and required but little treatment after.'

A form of remittent fever called *congestive*,—and styled by Dr. Dickson, 'a hideous and pestilential modification,'—prevails to a great extent over a large portion of our northern and north-western states, and is frequently terribly destructive. It commences often as a common intermittent, and the first paroxysm frequently attracts but little attention. After an interval of variable length, another rigor occurs, which may be prolonged for several hours, until reaction or death takes place. This is remarkable for the extreme coldness and death-like hue of the face and extremities. There is violent gastro-intestinal irritation, with incessant purging and vomiting. The discharges are often mixed with blood, and rarely with bile. Dr. Parry, of Indiana, says that they have 'the appearance of water, in which a large portion of recently killed beef has been washed.' There is but slight abdominal tenderness, but a sense of weight, and burning heat in the stomach are complained of. The thirst is intense, and unquenchable. The respiration is peculiar; it is described as consisting of 'a deep drawn double inspiration (or double sigh,) with one expiration;' the patient complains that he cannot get his breath. The pulse is small, thready, and frequent, beating from 120 to 140 in a minute; it sometimes becomes imperceptible for several hours before death, though generally, it is to be felt to the last. The body is bathed in a cold, clammy sweat, occasionally limited to the face and neck—the skin being of a livid hue and shriveled. There is usually excessive restlessness, the patient continually tossing about, and endeavouring to get out of bed. In many instances the brain is undisturbed, the intelligence remaining until death. In some cases there is severe cephalalgia and even delirium; and in others coma makes its appearance early in the second paroxysm. If no abatement in these symptoms occurs, death takes place in from twenty-four to sixty hours, the patient expiring in great agony. If, however, the remedies have acted, the restlessness diminishes, the skin dries, the pulse falls and becomes developed, and the body gradually attains its natural temperature. Dr. Wharton of Mississippi, observes that "this is a very slow process, as it often requires from twenty-four to forty-eight hours for the heat to travel from the knees to the extremities of the toes.' Dr. Boling asserts that 'notwithstanding the small and thready state of the pulse, in this variety of pernicious fever especially, the action of the heart will be found strong, as indicated by the loudness of its sounds, and the force of its impulse.'"

The following remarks by Dr. Shapter, on purgatives, deserve to be remembered, especially in those locations, and there are such, where a majority of the patients suffer from the complication of gastro-enteritis, in the course of the treatment.

"The disease of the general state of the system is to be obviated by the prompt administration of purgatives, in order to clear the primæ viæ from the morbid secretions found in the stomach and intestines; the reduction of the over excited heart's action by bloodletting, and the use of medicines of a diaphoretic nature. With the exception of some of the French writers, all practitioners agree on the necessity of administering purgatives; at the same time they are to be used with considerable judgment, as much mischief frequently accrues from their abuse. If violent and irritating cathartics are too unsparingly administered, they are very apt to set up an irritation in the mucous lining of the bowels, which is attended by consequences so imminent, as often to be the source of more serious alarm than the effects of the disease itself. There can be no greater mistake in the treatment of remittent fever, than the exhibition of a rapid succession of this class of medicines. They tend rather, by their local irritation, to increase the acrid nature of the secretions. At the same time it is absolutely necessary, especially at the onset of the fever, that effective evacuants should be administered; but those selected for this purpose should be but little irritating or drastic in their operation. The use of purging enemata will be found very useful adjuncts to the employment of purgatives, but they must by no means be solely relied on; and where there is much gastric irritation, and there are but few cases where there is none, a small quantity of the syrup of white poppies should be added, the addition tending very much to allay pain and to soothe the system."

The editor's remarks on the use of emetics, are very judicious, and agree with the opinions of Dr. Shapter on the same subject.

"Emetics are now generally abandoned in the treatment of this form of fever, it being found that they aggravate the gastric distress, which is usually so annoying. If, however, it is found necessary to empty the stomach at the commencement of an attack, the mildest means that will effect the object, should be resorted to."

The mode of using quinine in remittents is a question of much importance. And as there is some difference of opinion on this point, we give Dr. Clymer's views, which we believe will be found a correct guide to its employment.

"With regard to the administration of quinine some difference of opinion prevails. As a general rule, the more decided the remission, the greater its utility; in the inflammatory form, it is generally of little advantage. Where the remissions are well marked, or where there is a tendency to prostration, it is, on the contrary, of the highest value, and should be administered in doses of from five to ten grains, frequently repeated before the anticipated exacerbation."

The treatment for congestive remittent, is, like that for the intermittent type, supported by the authority of many American practitioners.

"The congestive form of remittent fever requires prompt and vigorous treatment. The chief indications are to procure reaction, and to prevent a recurrence of the paroxysm. To effect the first, sinapisms should be applied over the stomach, chest, between the shoulders, and on the extremities. Blisters are recommended by some practitioners as preferable, whilst others advise that they should replace the sinapisms, when these have commenced to excite irritation of the surface. Stimulants must be freely given internally, quinine, camphor, capsicum, &c. The quinine must be given in large doses, (grs. v. to x.,) at short intervals; it should be combined with capsicum or camphor, or if there is severe vomiting, the oil of turpentine may be substituted for the camphor. The evidence in favour of large doses of quinine repeatedly given in congestive fever, is ample and convincing. Calomel is generally combined with the above remedies, and is administered during the paroxysm, to the amount of fifteen or twenty grains."

With the matter added by the editor this is probably one of the best works we have on fevers. And especially adapted to the wants of the American physician.

